# Cognitive awareness in people with multiple sclerosis before and after neuropsychological assessment

**DOI:** 10.1007/s00415-025-13486-2

**Published:** 2025-11-05

**Authors:** P. T. Waskowiak, B. A. de Jong, B. M. J. Uitdehaag, A. Myronenko, H. E. Hulst, M. Klein

**Affiliations:** 1 Amsterdam UMC Location Vrije Universiteit Amsterdam, Medical Psychology, MS Center Amsterdam, Amsterdam Neuroscience, De Boelelaan 1118, Amsterdam, The Netherlands; 2 Amsterdam UMC Location Vrije Universiteit Amsterdam, Neurology, MS Center Amsterdam, Amsterdam Neuroscience, Amsterdam, The Netherlands; 3 Present Address: Faculty of Social Sciences, Institute of Psychology, Leiden University, Health, Medical and Neuropsychology Unit, Leiden, The Netherlands; 4 Leiden Institute for Brain and Cognition, Leiden, The Netherlands

**Keywords:** Cognitive awareness, Metacognition, Self-awareness, Cognitive impairment, Neuropsychological assessment, Multiple sclerosis

## Abstract

**Background:**

The association between subjective and objective cognitive functioning in people with multiple sclerosis (PwMS) is weak, making it difficult for clinicians to determine if referral for neuropsychological assessment is needed. We examined cognitive awareness in PwMS, its change after undergoing neuropsychological assessment, and its association with mood, fatigue, and objective cognitive functioning.

**Methods:**

PwMS were recruited as part of an observational study (Don’t be late!). Participants estimated their performance on the Minimal Assessment of Cognitive Function in MS (MACFIMS) battery before and after the assessment, relative to a demographically matched peer group. Participants were classified as overestimators, accurate estimators, or underestimators, based on discrepancies between subjective and objective percentile scores. Symptoms of mood and fatigue were assessed with the Hospital Anxiety and Depression Scale and the Modified Fatigue Impact Scale.

**Results:**

The sample included 228 PwMS (mean age = 48.39 ± 11.15 years; 70.2% female). Prior assessment, 123 participants (54%) overestimated, 70 (31%) accurately estimated, and 35 (15%) underestimated their cognitive performance. After assessment, fewer participants overestimated their performance (*N* = 89; 39%), while more accurately estimated (*N* = 89; 39%) or underestimated (*N* = 50; 22%) their performance. Fatigue and objective cognitive functioning predicted cognitive awareness at both time points (all *p* < 0.005); depression only before testing (*p* = 0.040), and anxiety was not a significant predictor (*p* > 0.417).

**Conclusion:**

About half of PwMS overestimate their cognitive performance before neuropsychological assessment. While task experience generally improves estimation accuracy, it also leads to increased underestimation in some PwMS.

## Introduction

Multiple sclerosis (MS) is a chronic autoimmune disease of the central nervous system, characterized by neuroinflammation and neurodegeneration [1]. In addition to a range of physical symptoms, up to 65% of people with MS (PwMS) have cognitive deficits, particularly in information processing speed and memory [2]. These cognitive limitations can severely impact relationships, employment, and health-related quality of life in PwMS [3–5]. Despite its impact, cognitive functioning is not routinely assessed in clinical practice and referral to a neuropsychologist largely depends on self-reported cognitive functioning [6]. Consequently, the ability to assess one’s own cognitive skills is crucial for the identification of cognitive impairment in this population [7].

At the same time, previous studies in PwMS have found that subjective cognitive functioning, often assessed via self-rated questionnaires, has only weak to moderate associations with actual performance on neuropsychological tests [7–9]. In fact, cognitive complaints in PwMS are more strongly associated with mood disturbances and fatigue than with objective cognitive performance [7, 9–12]. This suggests that self-reported cognitive limitations may primarily reflect psychological factors rather than actual cognitive deficits.

This discrepancy between subjective and objective cognitive functioning, raises the question of how much insight PwMS have into their own cognition. Cognitive awareness refers to an individual’s ability to accurately assess their own cognitive functioning and can be understood as the intersection of metacognitive knowledge and metacognitive experience [7]. Metacognitive knowledge involves stable, long-term beliefs about one’s cognitive abilities (e.g., “I have a poor memory”), while metacognitive experience reflects real-time judgments or feelings during cognitive tasks (e.g., “This test feels difficult”). These two components interact dynamically to shape cognitive self-evaluation [13]. Situated within the broader construct of self-awareness, which encompasses awareness of traits, emotions, and behaviours, cognitive awareness specifically pertains to insight into cognitive performance. In this study, cognitive awareness is defined as the accuracy with which individuals estimate their neuropsychological test performance.

Few studies have explored PwMS’ cognitive awareness on specific neuropsychological tests. For instance, Mazancieux et al. [14] found that individuals with relapsing–remitting MS (RRMS) tend to overestimate their performance, particularly on tasks where they also showed impairment [14]. Likewise, a small study with 18 PwMS and 16 healthy controls, Goverover et al. [15] reported that PwMS were significantly less accurate than healthy controls in estimating their own performance on a functional cognitive task (i.e., access the Internet to purchase airline tickets or cookies) [15]. Interestingly, this study also demonstrated that direct experience with the task improved awareness equally in both groups, suggesting that metacognitive experiences, in addition to metacognitive knowledge, needs to be considered within the context of cognitive awareness. The dynamic model of self-awareness [16] further supports this notion, assuming an interaction between the experience of performing a task and cognitive awareness.

However, research on metacognitive experiences in PwMS remains scarce, with existing studies focusing mainly on task-specific predictions. It therefore remains unclear how PwMS rate their overall cognitive functioning, which may be more informative for daily life functioning. In addition, most studies have not considered the actual presence or absence of cognitive impairment, which may also affect cognitive awareness. Finally, previous studies have typically small sample sizes and mainly included individuals with RRMS, limiting the generalizability of these findings to other MS subtypes [14, 15].

Understanding cognitive awareness in PwMS is essential for the early detection of cognitive impairment and the effectiveness of cognitive rehabilitation. Individuals with little awareness may fail to recognize cognitive deficits, reducing the likelihood of seeking support or mentioning it at their consultation with the treating neurologist. They may also fail to use compensatory strategies [14]. Moreover, improving cognitive awareness may not only facilitate timely interventions but also enhance self-assurance and psychological well-being in PwMS [17]. Therefore, the present study aims to determine (1) to what extent PwMS are aware of their overall cognitive functioning as assessed by comprehensive neuropsychological testing, (2) whether undergoing neuropsychological testing alters cognitive awareness in PwMS, and (3) to what extent depression, anxiety, fatigue and objective cognitive functioning predict cognitive awareness in PwMS.

## Methods

### Study design

This study is part of the larger Don’t be late! study, a research project aimed at the early identification of cognitive symptoms, postponing cognitive decline, and preventing early unemployment in PwMS in the Netherlands. For a full description of the project, the reader is referred to the study protocols [18, 19]. In the present study we used data from the first work package of the Don’t be late! study: a multicenter cross-sectional observational study [18]. The data for the present analyses were collected between July 2022 and March 2024.

### Participants

Participants had a confirmed MS diagnosis according to the McDonald 2017 criteria [20] and were under treatment at one of the participating hospitals in the Netherlands. They had no recent relapse, steroid treatment (last 6 weeks) or changes in disease modifying therapy (last 3 months). Participants who were diagnosed with another neurological or psychiatric disorder potentially influencing cognitive functioning, a history or current drug or alcohol abuse, or did not speak Dutch were excluded. For this study, all participants who had signed informed consent and completed at least five tests of the Minimal Assessment of Cognitive Function in MS battery [21] as well as both awareness measures (see below) were included in the present study.

### Measures

### Demographic and clinical characteristics

Demographic information including age, sex and educational level [22] were assessed. Additionally, information on MS subtype and disease duration was obtained. Disease severity was assessed with the telephone version of the Expanded Disability Status Scale (EDSS) [23, 24].

### Objective cognitive functioning

Objective cognitive functioning was measured with the Minimal Assessment of Cognitive Function In MS (MACFIMS) test battery assessing six cognitive domains most often affected in PwMS: information processing speed (Symbol Digit Modalities Test, SDMT [25], Paced Auditory Serial Addition Test, PASAT [26]), verbal and visuospatial learning and memory (Dutch Version of the California Verbal Learning, CVLT-II [27–29], Brief Visuospatial Memory Test-Revised, BVMT-R [30]), language and working memory (Controlled Oral Word Association Test, COWAT [31]), executive functioning (Delis-Kaplan Executive Function System sorting test, D-KEFS [32]), and visuospatial orientation (Judgment of Line Orientation Test, JLO [31]) [21]. We calculated a corrected composite percentile score for the MACFIMS by transforming the raw scores of each sub-test into a corrected z-score considering age (all, except for PASAT and D-KEFS), sex (all, except for BVMT-R), and educational level (all, except for D-KEFS) [33]. See section *Data analysis* for more details on this calculation. A higher objective percentile score indicated better objective cognitive functioning.

### Cognitive awareness

**Fig. 1:**
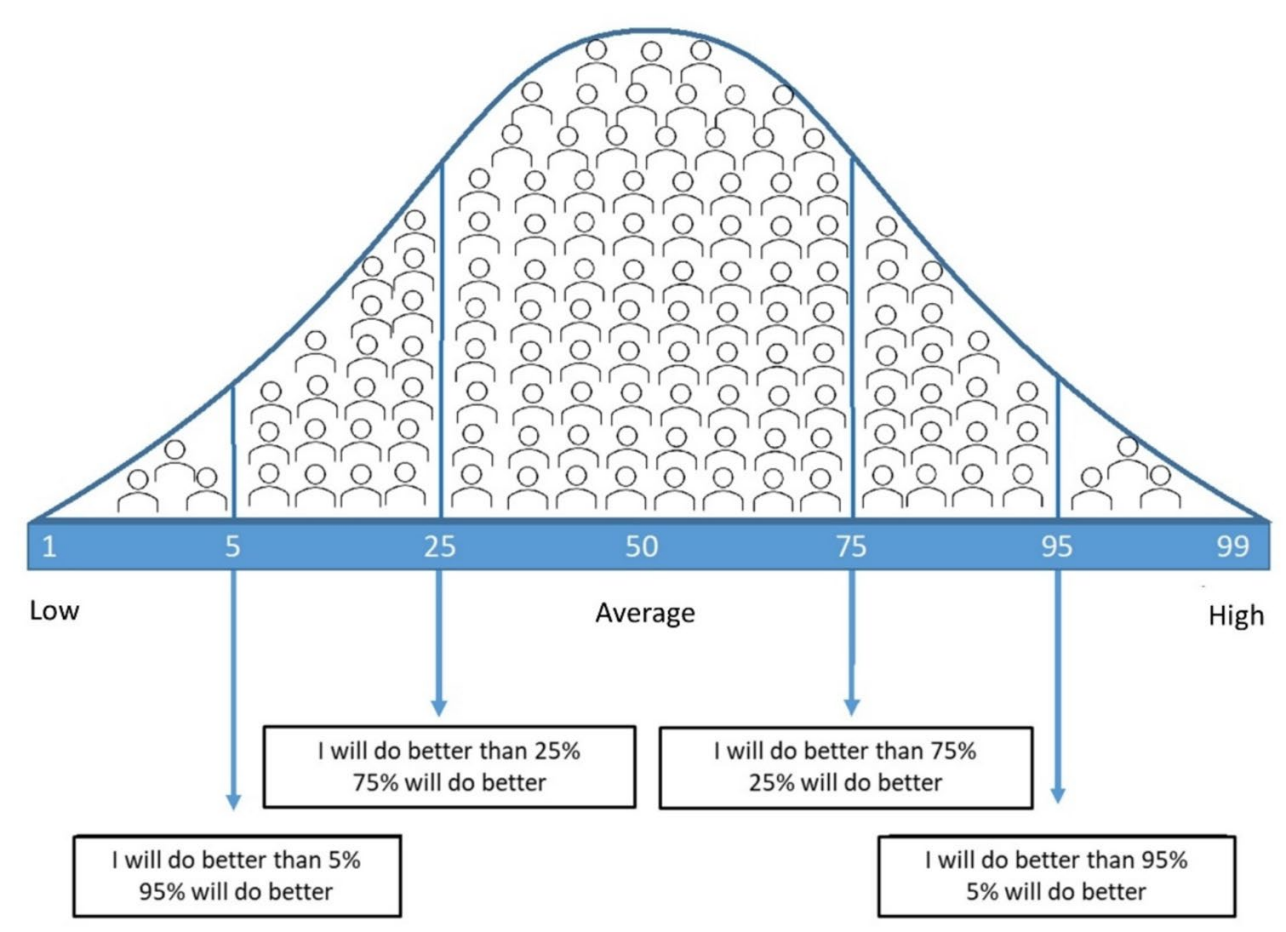
Normal distribution graph with explanations of the percentile scores

Cognitive awareness was assessed using the common-task common-metric approach developed by Rothlind and colleagues [34] for evaluating self-appraisal of neuropsychological performance [34]. This method has proven to be effective in evaluating cognitive awareness in healthy populations, as well as patients with Alzheimer’s Dementia and traumatic brain injury [34, 35]. In the present study, participants were asked to estimate their overall performance on the MACFIMS (see above) relative to a demographically matched peer group. This was done twice: just before and immediately after completing the test battery without having received any feedback on their performance. A normal distribution graph (inspired by [34]) with explanations of the percentile scores served as a visual aid for participants (see Fig. 1). If the explanation was unclear or participants were unfamiliar with the concept of a normal distribution, an example involving a familiar concept, such as height or shoe size, was given. The estimation resulted in two subjective percentile scores: one prediction (estimation before testing) and one postdiction (estimation after testing). In both cases, higher scores indicated better expected performance on the cognitive test battery.

### Mood and fatigue

The Hospital Anxiety and Depression Scale (HADS) was used to measure anxiety and depression [36]. Higher scores on the anxiety and depression subscales reflect greater severity of anxiety and depression symptoms, respectively. Fatigue was measured with the Modified Fatigue Impact Scale (MFIS) [37], with higher scores reflecting more severe fatigue.

### Ethics

The study was conducted according to the principles of the Declaration of Helsinki (2013) and in accordance with the Dutch Medical Research Involving Human Subjects Act (WMO). The Medical Ethical Committee of the Amsterdam UMC, Vrije Universiteit Amsterdam has reviewed and approved this study (METC 2021.0707, protocol version 2, 4 May 2022). All participants signed informed consent prior to participation.

### Procedure

Participants received a patient information letter regarding the study from their treating neurologist. Upon giving permission to be contacted by the researchers from Amsterdam UMC, participants underwent screening via telephone. Eligible participants were invited to the nearest participating hospital for the assessment. At the testing location, informed consent was signed. Then, several questions assessing the demographic and clinical characteristics (including the EDSS [23]) were asked. Before the MACFIMS was administered, participants received general instructions regarding the neuropsychological assessment and were then asked to predict their cognitive performance. Once the tests of the MACFIMS were completed, participants were asked to estimate their performance again (see *Measures* for more information on the assessment). The MACFIMS and awareness measures were performed in a paper–pencil manner. The HADS was administered at home via Castor EDC (Castor Electronic Data Capture v2024.1.2.0). The questionnaires were filled in within one week after the assessment at the hospital.

### Data analysis

First, z-scores for each MACFIMS subtest were calculated using regression-based norms, with age, gender, and education level as covariates [33]. These norm scores were based on studies conducted with healthy controls at Amsterdam UMC and Leiden University [38]. A percentile score was obtained by averaging the z-scores of each subtest and converting the result into a percentile.

Participants were classified as accurate, under-, or overestimators based on the difference between their subjective and objective percentile scores, with a predefined 10-point cut-off (established prior to data analysis). The difference was calculated by subtracting the objective percentile score from the subjective percentile score.

Before the main analysis, assumptions of normality, linearity, multicollinearity, and outliers were checked. Demographic and clinical characteristics across the groups of accurate estimators, underestimators, or overestimators were compared using Chi-square tests and univariate analysis of variance. Pearson’s correlation analysis was performed to assess the relationship between objective and subjective percentile scores. A 3 × 2 cross-table was created to visualize the distribution of accurate, under-, and overestimators before and after the MACFIMS battery.

Multinomial logistic regression was used to examine the effect of HADS anxiety and depression scores, MFIS scores, and objective percentile scores on the awareness group before and after (with the accurate group as the reference category). Additional exploratory logistic regression analyses were performed to assess the effect of the MFIS subscales on the awareness groups before and after. A post-hoc sensitivity analysis was conducted to test the robustness of the 10-point cut-off by adding and subtracting 2 points.

A significance level of *p* < 0.05 was used for all main analyses, and Bonferroni correction was applied for multiple comparisons. IBM SPSS Statistics 28 [39] and R studio [40] were used for the analysis.

## Results

### Demographic and clinical characteristics of PwMS

The sample included 228 PwMS, with the majority being female (70.2%) and having an average disease duration of 12.6 (*SD* = 9.1) years. See Table 1 for a full overview of the demographic and clinical characteristics. Performance on the subtests of the MACFIMS battery is presented in Table 2.

**Table 1:** Demographic and clinical characteristics of PwMS (*N* = 228)

Measure	
Age in years (*M,SD; range*)	48.4, 11.2; 21–67
Female	70.2%
Education^a^ (*median, IQR*)	6 (5–6)
Disease duration in years (*M,SD; range*)	12.6, 9.1; 0–42
EDSS (*median, IQR*)	3.5 (2.5–4.0)
MS type (RRMS/PMS/not specified)	172/45/11
DMT use (yes)	64.5%

*Note*. *MS* Multiple Sclerosis, *PwMS* People with MS, *M* Mean, *SD* Standard Deviation, *IQR* = interquartile range, *EDSS* Expanded Disability Status Scale, *RRMS* relapsing remitting MS, *PMS* primary and secondary progressive MS, *DMT* Disease Modifying Therapy

^a^Educational levels according to Verhage [41]: levels 1–5 completed average-level secondary education or lower; levels 6–7 completed high-level secondary education or university degree

**Table 2:** Performance on minimal assessment of cognitive function in MS test battery

MACFIMS (Sub) test	Z- score (M, SD)
CVLT-II direct (*n* = 228)	– 0.87 ± 1.07
CVLT-II delayed (*n* = 228)	– 0.42 ± 1.35
CVLT-II recognition (*n* = 228)	– 0.26 ± 1.0
BVMT-R direct (*n* = 228)	– 0.48 ± 1.13
BVMT-R delayed (*n* = 228)	– 0.58 ± 1.76
BVMT-R recognition (*n* = 228)	– 0.03 ± 0.86
SDMT (*n* = 226)	– 0.93 ± 1.01
JLO (*n* = 227)	– 0.20 ± 1.37
PASAT 3 s (*n* = 195)	– 0.17 ± 1.13
PASAT 2 s (*n* = 141)	– 0.04 ± 1.13
COWAT (*n* = 227)	– 0.95 ± 0.79
D-KEFS system sorting (*n* = 228)	– 0.27 ± 1.35

*Note*. *MACFIMS* Minimal Assessment of Cognitive Function in Multiple Sclerosis, *CVLT-II* Dutch Version of the California Verbal Learning Test, Second Edition, *BVMT-R* Brief Visuospatial Memory Test-Revised, *SDMT* Symbol Digit Modalities Test, *JLO* Judgement of Line Orientation Test, *PASAT* Paced Auditory Serial Addition Test, *COWAT* Controlled Oral Word Association Test, D-*KEFS* Delis-Kaplan Executive Function System sorting test, *M* Mean, *SD* Standard Deviation

### Research question 1: To what extent are PwMS aware of their overall cognitive functioning?

Before PwMS were tested with the MACFIMS battery, 123 participants (54%) overestimated, 70 participants (31%) accurately estimated, and 35 participants (15%) underestimated their cognitive performance. A statistically significant, weak positive correlation was found between the prediction percentile score and the objective percentile score, *r*(228) = 0.29, *p* < 0.001, indicating that PwMS who predicted higher scores also tend to actually score higher.

### Research question 2: To what extent does undergoing neuropsychological testing alter cognitive awareness in PwMS?

**Fig. 2:**
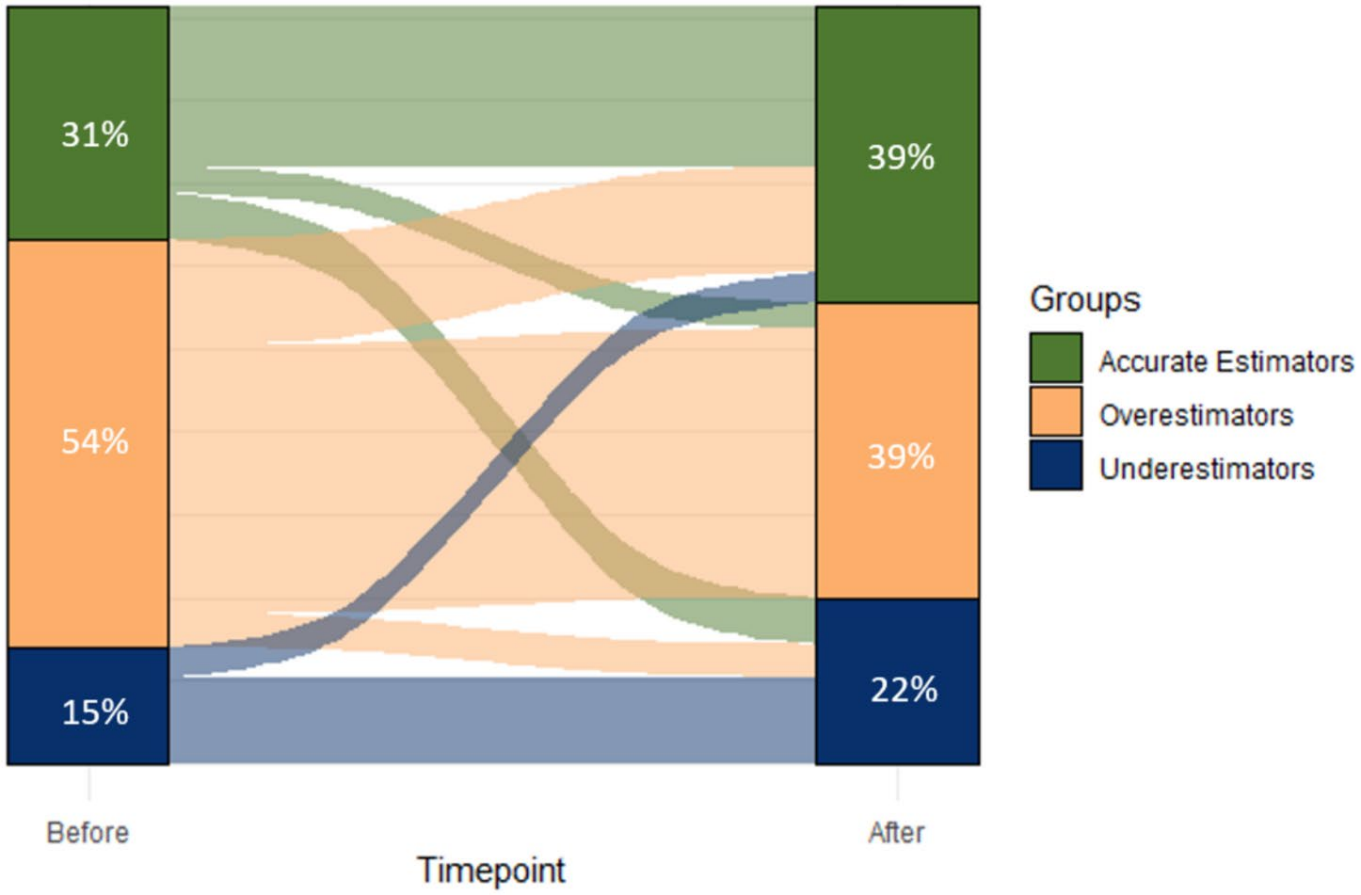
Cognitive awareness before and after neuropsychological assessment

After completing the MACFIMS battery, fewer participants overestimated their cognitive performance (*N* = 89; 39%; Δ =  – 34), while more participants accurately (*N* = 89; 39%; Δ =  + 19) or underestimated (*N* = 50; 22%; Δ =  + 15) their performance. Figure 2 illustrates the transition between groups of accurate estimators, underestimators, and overestimators before and after the MACFIMS battery. A significant association was found between the groups and categories, *χ*^*2*^(4, *N* = 228) = 127.12, *p* < 0.001, indicating a significant change in group membership. Interestingly, 26% of those who overestimated their performance before the MACFIMS accurately estimated it after the tests (see Fig. 2). In contrast, 20% of those who were accurate before the tests underestimated, and 11% overestimated their performance afterwards. Finally, we found a significant moderate positive association between the estimation percentile score after the MACFIMS and the objective percentile score, *r*(228) = 0.47, *p* < 0.001, suggesting an overall improvement in estimation accuracy from before (*r*(228) = 0.29) to after.

We found a statistically significant association between the groups of accurate estimators, underestimators, or overestimators before the assessment and the variable sex, *χ*^*2*^*(*2, *N* = 228) = 6.46, *p* = 0.042. Specifically, relatively few males underestimated their cognitive performance (standardized residual =  – 1.7), suggesting that men were less likely to underestimate themselves compared to women. No other significant differences were found between the groups of accurate estimators, underestimators, or overestimators before and after in terms of age, education, EDSS, disease subtype, or disease duration (all *p* > 0.077).

### Research question 3: To what extent does depression, anxiety, fatigue and objective cognitive functioning predict cognitive awareness in PwMS?

**Fig. 3:**
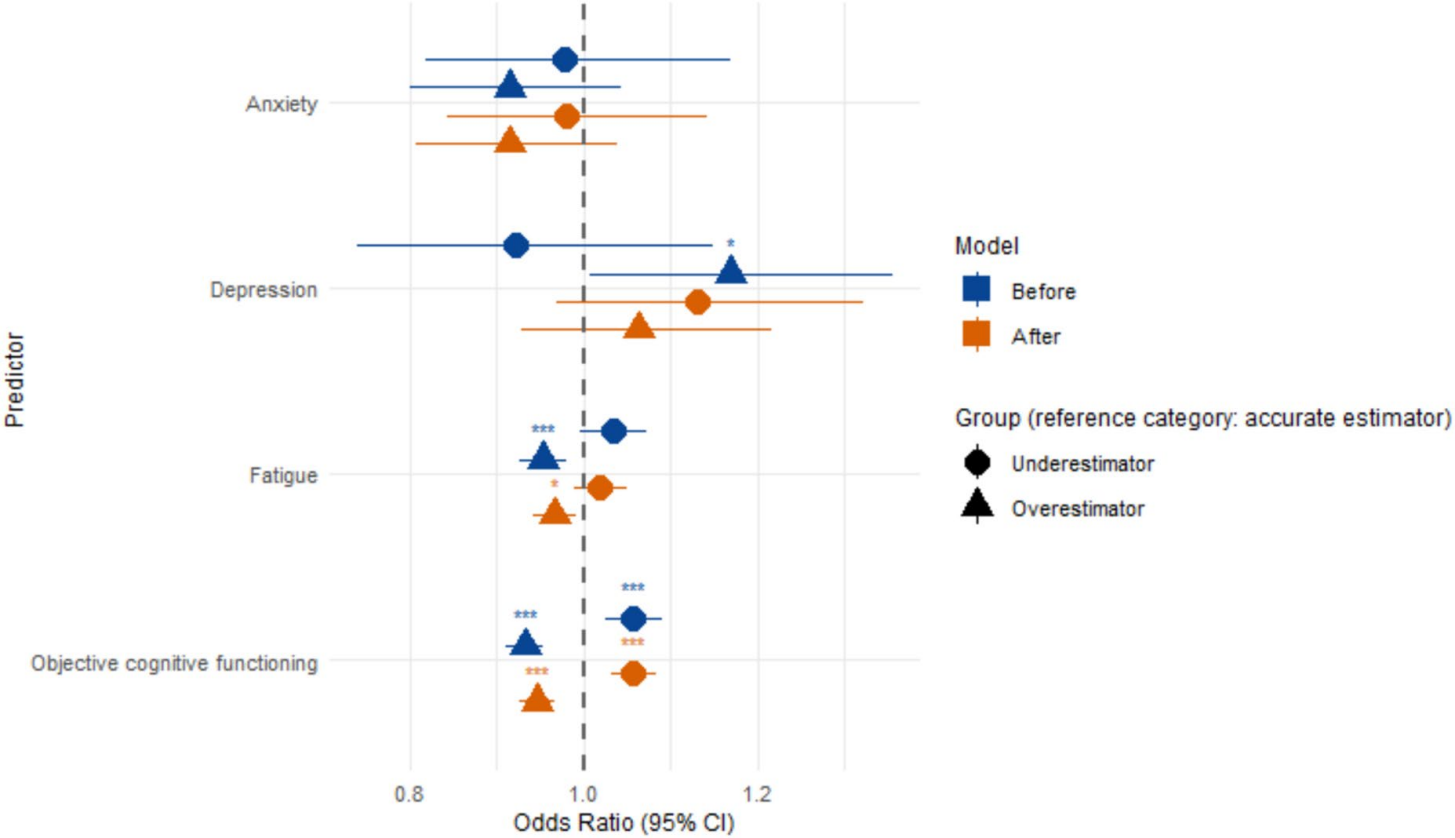
Predictors of cognitive awareness in PwMS before and after neuropsychological assessment. Note. *CI* Confidence interval. Underestimators and overestimators were compared to accurate estimators. Generally, the predictors range from lower on the left side to higher scores on the right side. The different scales of the predictors are not depicted in the graph. Analyses with and without outliers on the variables Anxiety and Depression yielded comparable results. *p < 0.05, ***p < 0.001

In our sample, overall depression (*M* = 3.83, *SD* = 3.52) and anxiety (*M* = 4.67, *SD* = 3.42) scores were relatively low. An overview of the depression, anxiety, fatigue, and objective percentile scores for all groups is presented in Table 3. Our multinomial logistic regression model showed that depression (χ^2^ = 6.456, *p* = 0.040), fatigue (χ^2^ = 20.524, *p* < 0.001) and objective cognitive functioning (χ^2^ = 98.516, *p* < 0.001) significantly predicted cognitive awareness prior undergoing neuropsychological assessment. Anxiety was not a significant predictor (*p* = 0.417). As can be seen in Fig. 3, compared to accurate estimators, underestimators were more likely to have higher objective cognitive functioning (Exp(B) = 1.057, *p* < 0.001), whereas overestimators tended to have higher depression scores (Exp(B) = 1.169, *p* = 0.039), lower fatigue (Exp(B) = 0.953, *p* < 0.001), and lower objective cognitive functioning (Exp(B) = 0.932, *p* < 0.001).

**Table 3:** Depression, anxiety, fatigue, and objective cognitive percentile scores for the estimation groups

Measure^a^	Timepoint^c^	Underestimators	Accurate estimators	Overestimators
HADS Depression (*M, SD)*	Before	4.50, 3.81 (*n* = 32)	4.95, 3.02 (*n* = 63)	4.57, 3.53 (*n* = 120)
	After	4.41, 3.87 (*n* = 46)	3.72, 3.38 (*n* = 81)	3.63, 3.46 (*n* = 88)
HADS Anxiety (*M, SD)*	Before	4.50, 3.81 (*n* = 32)	4.95, 3.02 (*n* = 63)	4.57, 3.53 (*n* = 120)
	After	5.15, 3.53 (*n* = 46)	4.94, 3.40 (*n* = 81)	4.18, 3.37 (*n* = 88)
MFIS Fatigue (*M, SD)*	Before	31.97, 15.93 (*n* = 33)	31.89, 16.30 (*n* = 63)	30.28, 18.29 (*n* = 120)
	After	33.41, 17.23 (*n* = 46)	31.95, 16.53 (*n* = 82)	28.88, 18.04 (*n* = 88)
Objective Percentile^b^ score (*M, SD*)	Before	58.32, 15.39 (*n* = 35)	43.57, 18.78 (*n* = 70)	25.58, 17.51 (*n* = 123)
	After	52.69, 18.66 (*n* = 50)	38.26, 20.17 (*n* = 89)	24.70, 17.19 (*n* = 89)

*Note*. *HADS* Hospital Anxiety and Depression Scale, *MFIS* Modified Fatigue Impact Scale, *M* Mean, *SD* Standard Deviation

^a^HADS and MFIS were completed by 215 and 216 participants, respectively

^b^The average of the Minimal Assessment of Cognitive Function in MS [21] test z-scores were converted to the objective percentile scores

^c^Importantly, values do not represent pre–post changes, but rather reflect scores of the estimation groups at both timepoints

After neuropsychological assessment, fatigue (χ^2^ = 10.968, *p* < 0.005) and objective cognitive functioning (χ^2^ = 84.964, *p* < 0.001), but not depression and anxiety (both *p* > 0.266), significantly predicted cognitive awareness. Compared to accurate estimators, underestimators were more likely to have higher objective cognitive functioning (Exp(B) = 1.057, *p* < 0.001) after neuropsychological assessment (see Fig. 3). Finally, overestimators were more likely to have lower fatigue (Exp(B) = 0.966, *p* = 0.010) and lower objective cognitive functioning (Exp(B) = 0.947, *p* < 0.001) compared to accurate estimators.

Additional exploratory analyses including the separate fatigue subscales, showed that only cognitive, but not physical or psychosocial, fatigue significantly predicted cognitive awareness after neuropsychological assessment (χ^2^ = 13.600, *p* = 0.001). Specifically, compared to accurate estimators, overestimators were more likely to report lower cognitive fatigue (Exp(B) = 0.888, *p* < 0.001).

## Sensitivity analysis

Sensitivity analysis with a cut-off score of 8 and 12 (instead of 10) yielded overall comparable results. Specifically, the multinomial logistic regression analyses with the alternative cut-off values showed that fatigue and objective cognitive functioning significantly predicted cognitive awareness before and after neuropsychological assessment. Comparable to the main analysis, depression was only a significant predictor of cognitive awareness before the neuropsychological assessment.

## Discussion

The present study investigated cognitive awareness in PwMS before and after neuropsychological assessment, and examined whether mood, fatigue, and objective cognitive performance predicts cognitive awareness. We found that half of PwMS overestimated their cognitive performance prior to testing. After the assessment, participants generally demonstrated improved cognitive awareness. However, a relatively larger group underestimated their cognitive abilities afterwards. Fatigue and objective cognitive performance were significant predictors of cognitive awareness before and after neuropsychological assessment, while anxiety and depression were not (consistently) associated with cognitive awareness in PwMS.

Although we found a higher percentage of overestimators (up to 54%) compared to the 2–28% reported by Mazancieux and colleagues [7], our findings remain generally consistent with these studies, which demonstrated a non-linear relationship between subjective and objective cognitive functioning in PwMS [7, 14]. In our study, we observed reduced awareness in individuals with both relatively low and relatively high objective cognitive functioning. Moreover, objective functioning emerged as the strongest predictor of estimation accuracy in our study. This pattern can be interpreted in light of the Dunning-Kruger effect [42], which suggests that individuals with lower cognitive performance may lack the insight (i.e., cognitive skills) to recognize their deficits, whereas high performers may be more sensitive to minor errors and therefore underestimate themselves [42]. Consequently, awareness patterns in PwMS mirror general cognitive biases, highlighting the need for objective testing rather than relying on self-report alone.

Importantly, participants showed significantly improved cognitive awareness after neuropsychological testing. This finding aligns with work by Goverover et al. who demonstrated that task experience improves cognitive awareness in both PwMS and healthy controls [15]. While their study used single functional tasks (e.g., access the Internet to purchase airline tickets), our results extend this observation to a broader, frequently used neuropsychological test battery [21]. Consequently, routine cognitive screening, beyond identifying impairment, might also support metacognitive insight in PwMS. Although participants in the present study did not yet receive feedback on their performance at the time of assessment, in clinical practice such feedback is an integral part of the testing process and may influence patients’ awareness of their cognitive functioning. Thus, whether repeated testing or structured feedback can further enhance cognitive awareness in PwMS remains a question for future research.

At the same time, however, it is important to note that relatively more people underestimated themselves after undergoing neuropsychological assessment. This shift may reflect increased uncertainty and could be related to affective symptoms such as depressed mood. A recent study in healthy controls suggests that individuals with elevated, but subclinical, anxiety and depression struggle to update their self-evaluations and show “*persistent underconfidence”* when evaluating their own performance [43]. Although we did not assess this mechanism directly, our results did not show a strong association between mood symptoms and cognitive awareness. This may be partly due to the relatively low average HADS scores in our sample, which were lower than those reported in previous MS samples [44]. Interestingly, we observed that participants with higher depressive symptoms were more likely to overestimate their performance prior to testing, a counterintuitive and underexplored result. One possibility is that mild depressive symptoms are associated with reduced introspection or self-reflection, while more severe depression might lead to specifically negative self-evaluation. However, as depressive symptoms were generally low in our sample, with limited variability, these interpretations remain speculative. Future research should explore whether depressive symptoms of varying severity differentially impact cognitive awareness in PwMS, ideally in samples that include a wider range of depressive symptomatology.

This study has several strengths, including a relatively large and heterogeneous sample, addressing a relatively underexplored area (metacognitive self-assessment in PwMS) especially before and after testing, use of a standardized and widely accepted cognitive battery (MACFIMS), along with validated mood and fatigue scales, strengthening internal validity. Nevertheless, there are some limitations to consider. First, the absence of a healthy control group limits our ability to determine whether the patterns we observed are MS-specific or reflect general tendencies in cognitive awareness. Second, we used an arbitrary cut-off score to classify estimation accuracy. Though, sensitivity analyses using slightly stricter and more lenient thresholds yielded comparable results, supporting the robustness of our findings. Third, the sample consisted of relatively young PwMS (mean age 48 years) and included fewer participants with progressive MS, which may limit the generalizability of our findings. Cognitive awareness might differ in older or progressive PwMS, who may have greater neurodegenerative burden and more pronounced cognitive impairment [45], potentially leading to reduced insight into their cognitive functioning. Finally, we did not assess participants’ reasoning behind their estimations. Several participants did spontaneously reflect on their reasoning during data collection, referring to factors such as age, fatigue, or caution in making too positive predictions. These observations align with qualitative findings by Yeandle et al., who reported that PwMS often feel uncertain whether cognitive difficulties reflect MS, aging, or normal variability, and may only become aware of changes when others point them out [5]. These observations highlight the potential value of incorporating qualitative methods to better understand how PwMS evaluate their own cognition, and which factors influence this process.

Our findings have both clinical and research implications. Since referral to neuropsychological assessment often relies on self-reported complaints, both overestimation and underestimation are clinically relevant. Overestimation may delay recognition and timely management of cognitive symptoms, whereas underestimation may lead to unnecessary worry or self-limiting behavior [17]. These observations support the rationale for establishing baseline and routine cognitive screening in MS care, as regular monitoring may help to detect subtle changes over time and enable timely intervention [4, 18]. Beyond these clinical considerations, our results also raise the question whether underestimation of cognitive performance might have predictive value. For instance, in Alzheimer’s disease, subjective cognitive impairment has been shown to predict later objective decline [46]. Although the underlying mechanisms differ, this may offer a useful conceptual parallel. In PwMS, underestimation might not only reflect a miscalibration between perceived and actual performance but could also indicate subtle or early decline that is not yet measurable with neuropsychological tests. Future longitudinal studies are needed to examine whether underestimation indeed represents an early marker of cognitive decline in MS. In conclusion, this study shows that half of PwMS overestimated their cognitive functioning prior to neuropsychological assessment, mirroring general cognitive biases. While testing overall helped improve cognitive awareness, underestimation also increased after testing in a subset of individuals, possibly reflecting increased uncertainty. Finally, fatigue plays a significant role in cognitive awareness, while mood effects are nuanced and may vary by severity or context. Our findings support the value of routine neuropsychological assessments and screening in MS care and suggest that improving awareness of cognitive functioning may be an additional benefit of the testing procedure. Future work should further examine the role of mood in cognitive awareness, and explore whether these awareness patterns are specific to MS.

## Data Availability

The anonymized data, not published in the article, will be shared upon reasonable request from a qualified investigator.
